# Phosphorylation regulates arginine-rich RNA-binding protein solubility and oligomerization

**DOI:** 10.1016/j.jbc.2021.101306

**Published:** 2021-10-19

**Authors:** Sean R. Kundinger, Eric B. Dammer, Luming Yin, Cheyenne Hurst, Sarah Shapley, Lingyan Ping, Sohail Khoshnevis, Homa Ghalei, Duc M. Duong, Nicholas T. Seyfried

**Affiliations:** 1 Department of Biochemistry, Emory University, Atlanta, Georgia, USA; 2 Department of Neurology, Emory University School of Medicine, Atlanta, Georgia, USA

**Keywords:** RNA-binding proteins, phosphorylation, posttranslational modifications (PTMs), mass spectrometry, protein interactions, SRSF2, SC35, ABC, ammonium bicarbonate, CIP, calf intestinal alkaline phosphatase, FA, formic acid, FDR, false discovery rate, LC, low complexity, LC-MS/MS, liquid chromatography coupled with tandem mass spectrometry, LLPS, liquid–liquid phase-separated, NCPR, net charge per reside, PTM, posttranslational modification, RBP, RNA-binding protein, RRM, RNA recognition motif, SRPK, SR protein kinase, TFA, trifluoroacetic acid, WGCNA, weighted gene correlation network analysis

## Abstract

Posttranslational modifications (PTMs) such as phosphorylation of RNA-binding proteins (RBPs) regulate several critical steps in RNA metabolism, including spliceosome assembly, alternative splicing, and mRNA export. Notably, serine-/arginine- (SR)-rich RBPs are densely phosphorylated compared with the remainder of the proteome. Previously, we showed that dephosphorylation of the splicing factor SRSF2 regulated increased interactions with similar arginine-rich RBPs U1-70K and LUC7L3. However, the large-scale functional and structural impact of these modifications on RBPs remains unclear. In this work, we dephosphorylated nuclear extracts using phosphatase *in vitro* and analyzed equal amounts of detergent-soluble and -insoluble fractions by mass-spectrometry-based proteomics. Correlation network analysis resolved 27 distinct modules of differentially soluble nucleoplasm proteins. We found classes of arginine-rich RBPs that decrease in solubility following dephosphorylation and enrich the insoluble pelleted fraction, including the SR protein family and the SR-like LUC7L RBP family. Importantly, increased insolubility was not observed across broad classes of RBPs. We determined that phosphorylation regulated SRSF2 structure, as dephosphorylated SRSF2 formed high-molecular-weight oligomeric species *in vitro*. Reciprocally, phosphorylation of SRSF2 by serine/arginine protein kinase 2 (SRPK2) *in vitro* decreased high-molecular-weight SRSF2 species formation. Furthermore, upon pharmacological inhibition of SRPKs in mammalian cells, we observed SRSF2 cytoplasmic mislocalization and increased formation of cytoplasmic granules as well as cytoplasmic tubular structures that associated with microtubules by immunocytochemical staining. Collectively, these findings demonstrate that phosphorylation may be a critical modification that prevents arginine-rich RBP insolubility and oligomerization.

RNA-binding proteins (RBPs) cooperatively engage both RNA and protein (1). RBPs frequently contain an RNA-binding domain, typically K-homology (KH), or RNA recognition motif (RRM) domains, which allow the RBP to achieve sequence-specific binding to target RNA molecules (2). Unbiased RNA interactome studies have identified many RBPs containing low-complexity (LC) domains that participate in both RNA and protein interactions (2, 3, 4, 5). LC domains are typically composed of a select few residues out of the entire amino acid code, giving rise to protein domains that are intrinsically disordered (6). However, LC RBPs exist in a dynamic continuum of native states that range from soluble monomers to liquid–liquid phase-separated (LLPS) granules to insoluble fibrils (7) *in vitro* and *in vivo* (8). These assembly states are believed to be influenced in large part by RNA molecules (9, 10) and posttranslational modifications (PTMs) (11, 12). Although the field is beginning to decipher a “molecular grammar” regulating LLPS (13), the conditions that give rise to irreversible aggregation are incompletely known.

Recently it has been discovered that the progression of several neurodegenerative diseases is promoted by the aggregation of RBPs (14, 15, 16, 17, 18, 19, 20, 21). Interestingly, LC domains are necessary for RBP LLPS and fibrillization (16, 19, 22, 23), processes found to be regulated by PTM. LC RBPs are commonly modified by reversible PTM in the physiological milieu (13, 24), yet in neurodegenerative disease phosphorylation PTMs increasingly occupy RBPs such as TDP-43 (25, 26, 27). It remains unclear whether phosphorylation is a trigger, or rather a consequence, of pathogenic RBP aggregation.

A major gap in our understanding of LC RBPs is our inability to accurately map site-specific phosphorylation levels. Recently our group used middle-down proteomic approaches to demonstrate that arginine-rich LC RBPs have high steady-state levels of PTMs, particularly phosphorylation (28). One such group of arginine-rich RBPs with high levels of phosphorylation is the serine-/arginine-rich (SR) splicing factor family of RBPs (29). This 12-member RBP family is known to contain at least one RRM RNA-binding domain (30) at the N-terminus and a C-terminal arginine-/serine-rich (RS) domain distinguished by an expanded tract of RS dipeptide motifs, a phosphomotif conserved from yeast to humans (3). The most extensively studied regulator of SR protein function is phosphorylation, primarily catalyzed by nuclear cdc2-like kinases (CLKs) (31) and cytoplasmic SR protein kinases (SRPKs) (32, 33, 34). Phosphorylation regulates nearly every facet of SR protein function, including splicing (35), coupling to sites of active transcription (36, 37), subcellular localization (38, 39, 40), nuclear speckle compartmentalization (31, 32, 41) and binding partner selection and affinity (30, 39, 42, 43). Importantly, it is not fully understood whether excessive, or rather, insufficient phosphorylation alters the stability of SR proteins.

Our group (28) and others (40, 44, 45, 46) suggest that SR proteins may increasingly bind together and aggregate when insufficiently phosphorylated. Importantly, SR proteins and proteins that harbor homologous domains can aggregate under native conditions (47). Collectively, these data support a hypothesis that dephosphorylation would result in SR proteins becoming insoluble, as well as those RBPs with SR-like LC domains.

Here, we sought to understand the role of phosphorylation in regulating RBP solubility. We enriched for RBPs by biochemical fractionation from mammalian cell lines and incubated with calf intestinal alkaline phosphatase (CIP), which catalyzes the removal of phosphate PTMs from proteins (48). We conducted liquid chromatography coupled with tandem mass spectrometry (LC-MS/MS) on detergent-soluble and -insoluble pellet fractions of dephosphorylated and mock-treated nucleoplasm extracts and used a network-based approach to identify groups of RBPs that exhibited similar solubility changes that were regulated by phosphorylation. Importantly, we found that SRSF2 and related SR proteins coaggregated to the insoluble fraction, while other nuclear RBPs such as TDP-43 did not. Moreover, we found that phosphorylation regulates SRSF2 assembly states *in vitro*. Finally, we show that pharmacological SRPK inhibition in cells results in an increase in the number of cells harboring cytoplasmic SRSF2 granules as well as filamentous-like structures that colocalize with microtubules. Collectively, this work reinforces phosphorylation as an important regulator of SR protein solubility and structure and suggests that phosphorylation may be a preventative cellular mechanism against arginine-rich RBP aggregation.

## Results

### Phosphorylation prevents SRSF2 aggregation

Here, we use SRSF2 as a paradigm to study the regulation of arginine-rich RBP solubility, structure, and morphology by phosphorylation. In SRSF2, the arginine-/serine-rich (RS) domain is highly phosphorylated (28), a region with high probability of intrinsic disorder (Fig. S1*A*) (49, 50). We incubated lysates containing recombinant SRSF2-myc, a known phosphoprotein, with CIP, which corresponded to a lower-molecular-weight SRSF2 band (Fig. S1*B*) by SDS-PAGE, suggesting substantial dephosphorylation of SRSF2 (51, 52). To further validate dephosphorylation of SRSF2, we immunoblotted with an antibody raised against the C-terminus of SRSF2 that preferentially labels hypophosphorylated SRSF2 (*hypo*SRSF2) (53, 54, 55) (Fig. S1*C*) and again observed increased migration of SRSF2. Furthermore, we saw an increase in *hypo*SRSF2 immunoreactivity, demonstrating that SRSF2 is indeed dephosphorylated.

**Figure 1:**
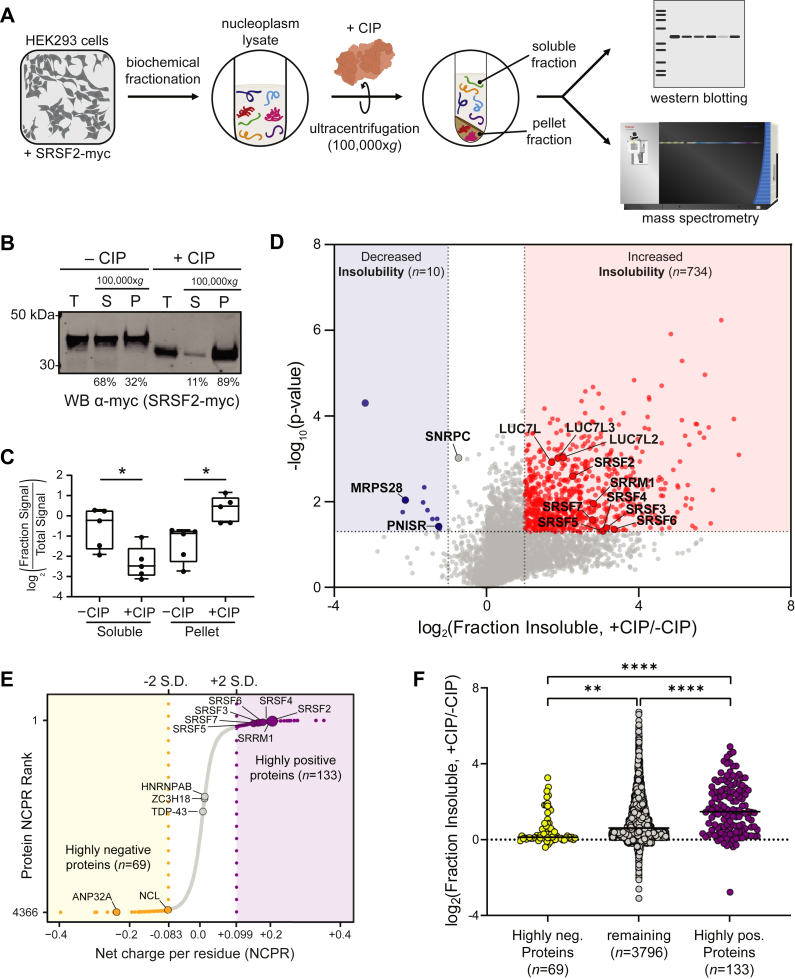
**Phosphorylation regulates arginine-rich RNA-binding protein solubility.***A*, sample preparation and proteomic workflow. Nucleoplasm extracts of HEK293 cells expressing recombinant SRSF2-myc protein were incubated with either calf intestinal phosphatase (+CIP) or distilled water (−CIP) at 37 °C for 1 h. Following this, samples were ultracentrifuged at 100,000*g* for 1 h. The soluble and insoluble pellet fractions were desalted and run by either western blot or liquid chromatography coupled with tandem mass spectrometry. *B*, prespin input (total, *T*), supernatant (soluble, *S*), and insoluble pellets (*P*) were run by SDS-PAGE and western blotted for myc. The average percent soluble (*sol./(sol. + insol.)*] and insoluble (in*sol./(sol. + insol.)*] values were calculated for five biological replicates and displayed below the representative western blot. *C*, band densitometry of soluble and pellet fraction log_2_-transformed SRSF2-myc band intensities normalized to the total signal in −CIP and +CIP conditions (five biological replicates; Soluble *p* value = 0.0123; Pellet *p* value = 0.0372; two-tailed paired *t* test). *D*, differential abundance of proteins in the soluble fractions. Fold-change, displayed on the x-axis, was the log_2_ value for fraction of signal that was insoluble [insoluble/(insoluble + soluble)] for the pairwise comparison +CIP/−CIP. The t-statistic (−log_10_(*p*-Value)) was calculated for all proteins and displayed on the y-axis. Insoluble-enriched proteins were highlighted in *red* (log_2_(fold change) ≥1, *p* value < 0.05) and proteins depleted from the insoluble fractions upon dephosphorylation were highlighted in *blue* (log_2_(fold change) ≤ −1, *p*-Value < 0.05) *squares*, respectively. *E*, an S-graph ranking each protein by the NCPR value. Proteins with NCPR values two standard deviations (2 S.D.) below or above the mean NCPR value of all proteins (0.0076) split the proteome into three groups: highly negative (*yellow*, < −0.083, *n* = 69), remainder (*gray*, −0.083 < × < +0.099, *n* = 3796) and highly positive (*purple*, *n* = 133, > +0.099). RNA-binding proteins in each group are highlighted, including SRSF2 and other SR proteins, which rank among the highest NCPR value proteins in the proteome. *F*, grouped scatter plot of the log_2_-transformation of the difference of fraction insolubility values of +CIP and mock conditions values for high negative proteins (*yellow*), highly positive proteins (*purple*) and remaining proteins (*gray*). Fraction Insolubility values were compared between groups (unpaired *t* test, ∗*p* < 0.05, ∗∗*p* < 0.01, ∗∗∗*p* < 0.001, ∗∗∗∗*p* < 0.0001).

We then asked whether phosphorylation regulates the solubility of SRSF2. Detergent-soluble (S) and -insoluble pelleted (P) fractions were isolated following mock (−CIP) or phosphatase (+CIP) treatment (Fig. 1*A*). We resolved equal amounts of total (T), soluble (S), and pelleted (P) fractions by SDS-PAGE and immunoblotted for SRSF2-myc (Fig. 1*B*). Labeling of each fraction with the *hypo*SRSF2 antibody confirmed that the pellet fraction was enriched with *hypo*SRSF2 species (Fig. S1*D*). While phosphorylated SRSF2-myc was primarily soluble (68% of total) in the mock condition, dephosphorylated SRSF2-myc significantly decreased in solubility, enriching to the detergent-insoluble pellet fraction (89% of total, Fig. 1*C*). This suggests that in addition to SRSF2, similar arginine-rich RBPs or groups of RBPs may experience altered solubility following dephosphorylation. We next sought to globally identify and quantify RBPs that aggregate following dephosphorylation.

### Proteomics reveals RBPs that aggregate following dephosphorylation

Following dephosphorylation, the soluble and insoluble (*i.e.*, pellet) samples were analyzed by label-free quantitative proteomics, using liquid chromatography coupled with tandem mass spectrometry (LC-MS/MS) in biological quadruplicate (Fig. 1*A*, Table S1). Notably, dephosphorylation did not induce global aggregation of the nuclear proteome, as insoluble pellet fraction protein concentrations were unchanged after phosphatase incubation (Table S2). Following database search and removal of proteins with >50% missing values and single peptide identifications, we identified 4120 unique proteins (Table S3 and S4).

To discover proteins with the largest change in solubility following dephosphorylation, we calculated the log_2_ fold differences of fraction insoluble values between phosphatase and mock treatments and visualized this as a volcano plot (Fig. 1*D*, Tables S4 and S5). Proteins were highlighted with increased or decreased insolubility if at least a twofold increase or decrease in fraction insoluble values, respectively, was observed with a *p* value less than 0.05 (two-tailed paired *t* test). Relatively few proteins (*n* = 10) enriched to the soluble fraction following dephosphorylation. In contrast, many more proteins (*n* = 734) experienced increased aggregation following dephosphorylation. To ask whether serine-/arginine-rich (SR) proteins were enriched within this group, we performed a homology search of the RS domain of SRSF2 using the protein BLAST tool. We identified several proteins with high homology to the RS domain of SRSF2 (*n* = 193), including other SR proteins (SRSF3/4/5/6/7), as well as the SR-like proteins SRRM1 and LUC7L3 (Fig. S2*A*, Table S6). Using a one-dimensional hypergeometric Fisher’s exact test (FET) analysis, we concluded that the SR/SR-like group was significantly enriched to the list of proteins that experienced significantly decreased solubility following dephosphorylation (BH-corrected *p* value = 0.0132) (Fig. S2*B*). These observations suggest that phosphorylation is an important PTM that regulates the solubility of SRSF2, as well as the solubilities of similar arginine-/serine-rich RBPs.

### Arginine-/lysine-rich RNA-binding proteins with positive net charge preferentially aggregate following dephosphorylation

Phosphorylation significantly alters the net charge of a protein, adding a −2 charge with each phosphorylated residue at physiological pH (56). We hypothesized that proteins with highly positive net charge (high densities of arginine/lysine) may be predisposed to aggregate when not sufficiently phosphorylated. We plotted the distribution of net charge per reside (NCPR) of the nucleoplasm proteome sequenced and highlighted those proteins that were two standard deviations below (<−0.083; *yellow*) or above (>+0.099; *purple*) the mean NPCR (Fig. 1*E*). Interestingly, 11/12 members of the SR protein family surpass the positive NCPR threshold, with SRSF2 being the most positively charged overall. The SR RBP family has an extremely high positive average net charge, relative to other RBP families (Table S8).

Using the aforementioned NCPR criteria to split our proteome into groups of low, middle, and high NCPR, we asked whether higher intrinsic NCPR values conferred increased susceptibility to protein aggregation following dephosphorylation. Indeed, we saw a stepwise increase in average fraction insolubility values with increasing NCPR value (Fig. 1*F*). When compared with the group of proteins with low NCPR values (*n* = 69, <−0.083), middle-charged proteins (*n* = 3796, −0.083 < × < +0.099) had a significantly increased average fraction insolubility value (0.89 *versus* 0.436, respectively). Moreover, the high NCPR group (*n* = 133, > +0.099) had the highest fraction insolubility value (1.56), which was significantly increased when compared with both the middle-charged and low-charged groups. These data suggest that proteins with a high NCPR (>+0.099) are highly susceptible to increased aggregation following dephosphorylation. SRSF2, as well as SRSF3/4/5/6/7, all surpassed the +0.099 NCPR threshold and became highly insoluble following dephosphorylation. We conclude that phosphorylation may be an important mechanism to regulate the solubility of proteins with high concentrations of arginine and lysine, among which are many nuclear RBPs involved in splicing.

### Systems analysis identifies modules of proteins with solubility impacted by phosphorylation

We hypothesized that if we applied systems biology approaches to the protein abundance data we collected, we could discover groups of structurally similar proteins with shared biology that may coaggregate when not sufficiently phosphorylated. To test this, we performed Weighted Gene Correlation Network Analysis (WGCNA) (57) to group proteins with highly correlated soluble and insoluble fraction abundance patterns. To define functionally divergent protein groups, we plotted a dendrogram that was segregated by hierarchical clustering into modules of related proteins (Fig. S3). The network reduced our proteome into 27 modules [rank ordered by size, M1 (largest) – M27 (smallest)] each assigned a representative color (Table S7). Each module was classified by the strength of associations to GO terms linked to discrete and generalizable cellular functions.

**Figure 2:**
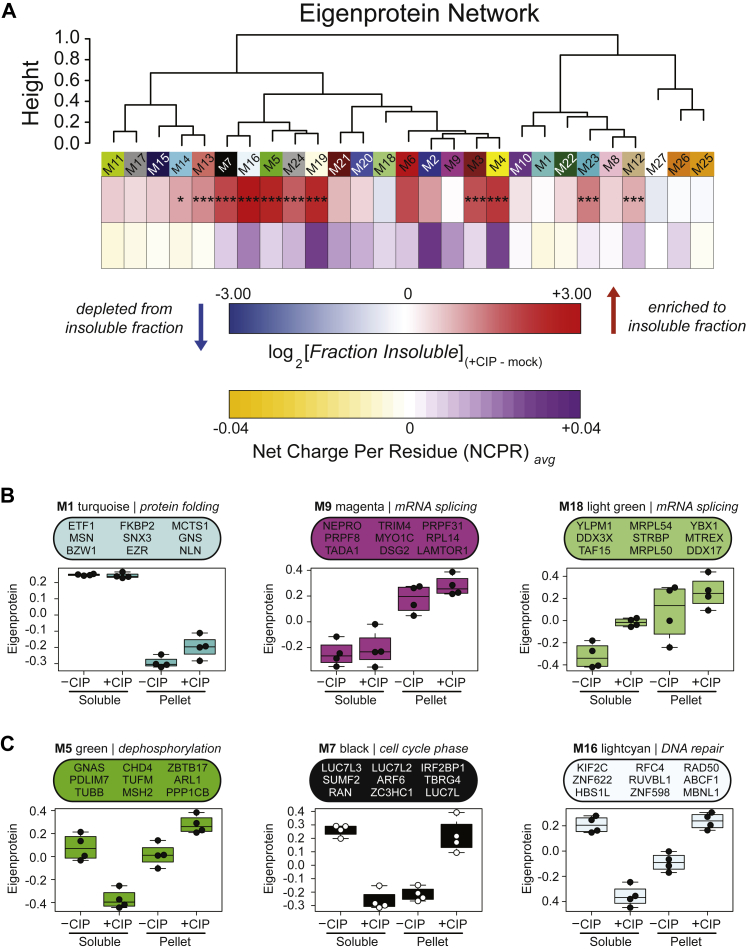
**A correlation network approach groups proteins into modules with discrete gene ontologies and solubility patterns in response to dephosphorylation.***A*, protein modules were clustered to assess module relatedness based on correlation with abundances in detergent-soluble and -insoluble pellet fractions in −CIP and +CIP conditions. Log_2_ values for fraction of signal that was insoluble [insoluble/(insoluble + soluble)] for the pairwise comparison +CIP/−CIP displayed as a heatmap for each module and compared with the average net charge per residue (NCPR) of all members of that module, also displayed as a heat map. Significance of enrichment to top 734 insoluble proteins displayed as *asterisks*, determined by one-dimensional hypergeometric Fisher’s exact test (FET, BH corrected; ∗*p* < 0.05, ∗∗*p* < 0.01, ∗∗∗*p* < 0.001). Modules with positive average NCPR are colored *purple* while those with negative average NCPR are colored *yellow*. *B* and *C*, eigenprotein abundance *box* and whisker plots of selected modules. The farthest data points, up to 1.5 times the interquartile range away from *box* edges, define the extent of whiskers (error bars). *B*, protein modules that are unchanged in solubility phosphorylated or dephosphorylated. *C*, selected modules with increased insolubility abundances following dephosphorylation.

To understand which module of proteins experienced the greatest alterations in solubility following dephosphorylation, we performed a one-dimensional hypergeometric FET for enrichment within each module of those proteins with the most increased insolubility following CIP treatment (*n* = 734) (Fig. 2*A*). Several key modules (M5, M7, M16) were enriched with proteins that aggregated following dephosphorylation. To identify modules with differences in solubility changes, eigenprotein values were plotted according to protein fraction and treatment condition (Fig. 2, *B* and *C*). Eigenproteins are defined as the first principal component of a module and serve as a representative, weighted module expression profile. As expected, some modules did not experience appreciable solubility changes following dephosphorylation. Among these were the soluble module M1 (protein folding) and insoluble module M9 (mRNA splicing), both unchanged in solubility profile after dephosphorylation (Fig. 2*B*). Other modules, however, including M5 (dephosphorylation), M7 (cell cycle phase), and M16 (DNA repair) represented a prominent group of modules comprised of proteins that are soluble when phosphorylated, yet aggregate upon dephosphorylation (Fig. 2*C*). The three members of the SR-like LUC7L family were each hub proteins of the M7 module, which experienced a large decrease in solubility. SRSF2 was a member of the M16 module, which interestingly also contained several cytoskeletal components (Table S7). Given the robust insolubility of individual modules following dephosphorylation, we sought to validate individual proteins within these modules that significantly changed in solubility as well.

### Confirmation of hub protein solubility changes following dephosphorylation

**Figure 3:**
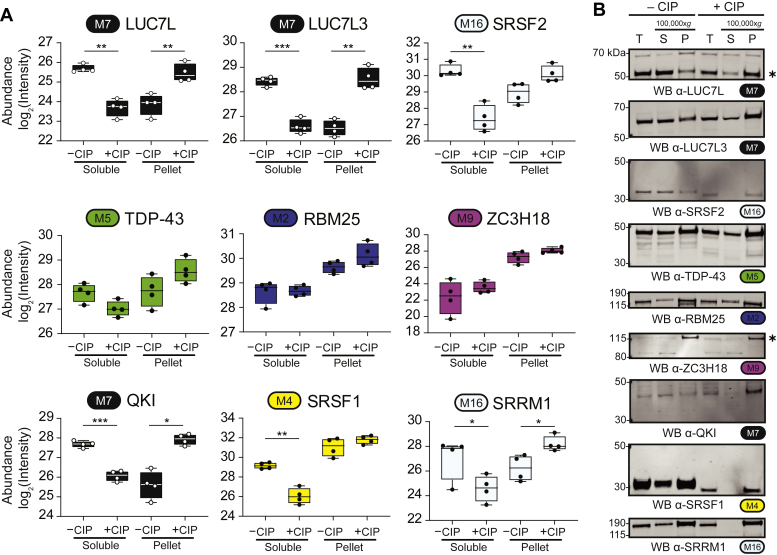
**RNA-binding proteins have variable abundance patterns in soluble and pellet fractions following dephosphorylation.***A*, *box* and whisker plots of mass spectrometry abundance measurements (*n* = 4) of RNA-binding proteins in soluble and pellet fractions in −CIP and +CIP conditions. (two-tailed paired *t* test; ∗*p* < 0.05, ∗∗*p* < 0.01, ∗∗∗*p* < 0.001). *Module number* and *color* are indicated next to each gene symbol. Whiskers range from min to max values. SRSF2 was significantly depleted from the soluble fraction while nearly significantly enriched to the pellet fraction (Soluble *p* value = 0.0063; Pellet *p* value = 0.0518). *B*, Western blot validation of solubility changes of well-described RNA-binding proteins. Modules, colored accordingly, are paired with the protein name.

To examine the effect of dephosphorylation on the solubility of individual RBPs, we plotted the mass spectrometry protein abundance measurements (Fig. 3*A*) and compared with immunoblot signals of select endogenous proteins from total, soluble, and pelleted fractions (Fig. 3*B*). The classical SR proteins SRSF1 and SRSF2 were depleted from the soluble fraction, which was verified by western blot. LUC7L and LUC7L3, both hubs (*i.e.*, most correlated to the module eigenprotein) of the M7 “cell cycle phase” module, were significantly depleted from soluble fractions and enriched to insoluble pellet fractions. Other nuclear RBPs in HEK293 nucleoplasm lysates including TDP-43 (M5), RBM25 (M2), and ZC3H18 (M9), however, showed no significant solubility changes following dephosphorylation. It should be noted that some RBPs harbor relatively few phosphorylation PTMs in the absence of stress conditions (*e.g.*, TDP-43), demonstrated by the lack of mobility change following dephosphorylation (28). Nevertheless, mass spectrometry examination of soluble and pellet fractions revealed global RBP solubility changes in response to phosphatase coincubation, revealing RBPs susceptible to destabilization following dephosphorylation.

### Phosphorylation decreases NCPR and regulates the oligomerization of arginine-rich SRSF2

**Figure 4:**
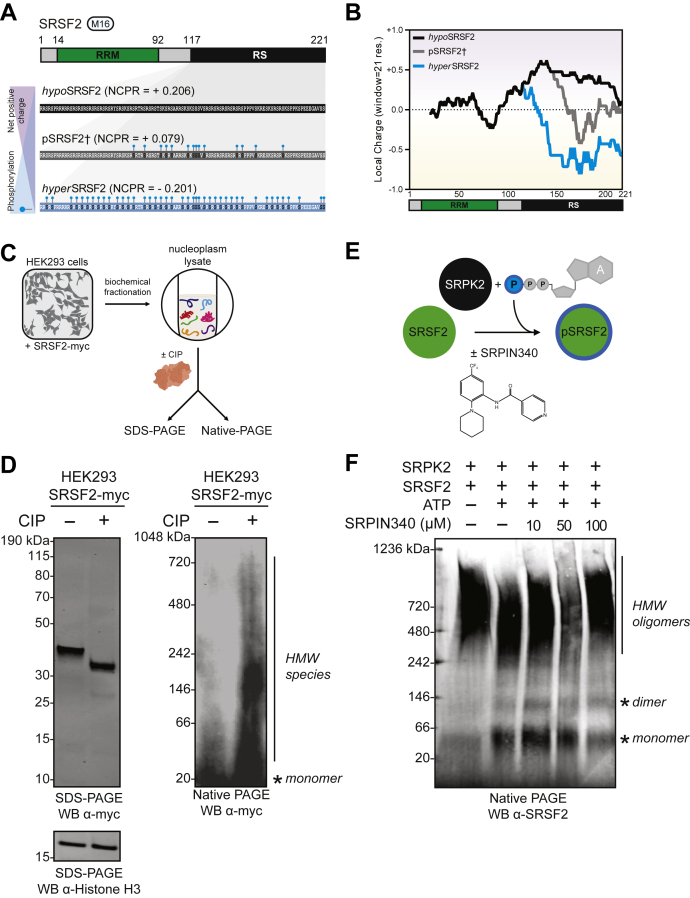
**SRSF2 net charge andoligomerization, respectively, increase substantially with dephosphorylation.***A*, SRSF2 net charge per residue (NCPR) calculated according to phosphorylation state, *i.e.*, no phosphorylation (*hypo*SRSF2), phosphorylation sites previously observed by middle-down mass spectrometry (pSRSF2†, Kundinger & Bishof *et al.*, 2020) or full phosphorylation (*hyper*pSRSF2). Phosphorylation sites represented by *turquoise ball* and *stick*. *B*, line plots of average charge density (window = 21 residues) from C-terminus to N-terminus of SRSF2 that is either nonphosphorylated (*black*), observed phosphorylation by MS (*gray*, Kundinger & Bishof *et al.*, 2020) or fully phosphorylated (*turquoise*) in the RS domain. The SRSF2 protein map is included below the line plot, with the RNA-recognition motif (RRM) domain (*green box*) and RS domain (*black box*) annotated. *C*, nucleoplasm extracts of HEK293 cells expressing recombinant SRSF2-myc protein were incubated with either calf intestinal alkaline phosphatase (+CIP) or distilled water (−CIP) at 37 °C for 1 h. Following this, samples were split and analyzed by both denaturing and nondenaturing native PAGE and western blotted for myc (*n* = 3). *D*, by denaturing SDS-PAGE (*left*), CIP-treated SRSF2-myc has increased electrophoretic mobility. Equal loading is demonstrated by Histone H3 labeling. Immunoblotting for SRSF2-myc after nondenaturing *Blue* native PAGE (*right*) identifies various dephosphorylated SRSF2 species not observed in mock-treated samples, including monomer (∼37 kDa, *asterisk*) and high-molecular-weight (HMW) species. *E*, diagram of *in vitro* kinase reaction using kinase SRPK2 (*black*) and substrate SRSF2 (*green*). Both SRPK2 and SRSF2 were expressed and purified from *E. coli* and were mixed in the presence (+) or lack thereof (−) ATP and SRPK inhibitor (SRPIN340) and incubated at 30 °C for 30 min. Phosphorylated SRSF2 was represented by a *turquoise ring*. *F*, the *in vitro* reaction was separated by nondenaturing native PAGE, which was transferred and western blotted for SRSF2. Monomeric (*asterisk*) and high-molecular-weight (HMW) oligomeric (*line*) species were unequally observed in the − and + ATP and different SRPIN340 concentration conditions by nondenaturing native PAGE.

With no consideration of PTMs, SRSF2 is among the most positively charged proteins in the entire proteome (Table S8). In a theoretical exercise of how phosphorylation changes the net charge of the SRSF2, we calculated the average SRSF2 NCPR (Fig. 4*A*) and local charge density in a 21 residue sliding window range (Fig. 4*B*) assuming three separate states: no phosphorylation (*hypo*SRSF2), MS-observed phosphorylation (pSRSF2†) (28), and full phosphorylation (*hyper*pSRSF2) (Table S9). Although substantially positively charged within the RS domain, SRSF2 adopts increasing negative charge with increasing phosphorylation, such that the RS domain becomes net negatively charged at full phosphorylation occupancy. As we observed increased SRSF2 aggregation upon dephosphorylation, we asked whether phosphorylation could similarly regulate the oligomerization of recombinant SRSF2.

Given the aggregation we observed for SRSF2, we hypothesize that a critical structural change occurs as a result of dephosphorylation. To test this, we analyzed phosphorylated and dephosphorylated lysates containing SRSF2-myc by both denaturing SDS-PAGE and nondenaturing Blue native PAGE followed by western blotting for the myc-tag of recombinant SRSF2 (Fig. 4, *C* and *D*). In contrast to SDS-PAGE, which separates proteins under denaturing conditions, native PAGE resolves native protein masses in high-molecular-weight oligomeric states as well as protein complexes formed by physiological protein–protein interactions (58). The mock-treated SRSF2-myc sample had a recognizable monomer species band at ∼37 kDa, whereas dephosphorylated SRSF2 exhibited monomer as well as high-molecular-weight oligomeric species (Fig. 4*D*). These data suggest that dephosphorylation leads to high-molecular-weight oligomeric SRSF2 structures, although in complex cellular lysate we could not determine whether the high-molecular-weight species are due to phosphorylation-dependent changes alone on SRSF2 or in part by protein–protein interactions as well.

To directly demonstrate how SRSF2 structure changes with phosphorylation, we performed an *in vitro* kinase reaction using purified SR protein kinase 2 (SRPK2) and SRSF2 substrate and analyzed reactions by nondenaturing native PAGE (Fig. 4, *E* and *F*) (59). As proof of principle, we confirmed that SRPK2 robustly phosphorylates SRSF2 substrate *in vitro* using radiolabeled ATP (Fig. S4). Both SRPK2 and SRSF2 were expressed and purified in *Escherichia coli*, an organism with a low abundance of phosphoproteins and only three known Ser/Thr-directed kinases (60, 61). We then separated these reaction products by nondenaturing native PAGE and found that unphosphorylated SRSF2 formed high-molecular-weight oligomers, with a prominent high-molecular-weight smear between 480 and 1000 kDa (Fig. 4*F*). In contrast, SRPK2-phosphorylated SRSF2 exhibited a decreased molecular weight range of oligomeric species (242–900), as well as a distinct monomer band at ∼50 kDa. Reactions incubated with increasing concentrations of SRPIN340, an ATP-competitive selective SRPK inhibitor (62), yielded a dose-dependent recovery of high-molecular-weight oligomer species along with the depletion of the SRSF2 monomer band, demonstrating that phosphorylation directly regulates the oligomerization state of SRSF2.

### SRPK inhibitor SRPIN340 decreases SR protein phosphorylation and increases SRSF2 cytoplasmic mislocalization and granule and tubule formation

**Figure 5:**
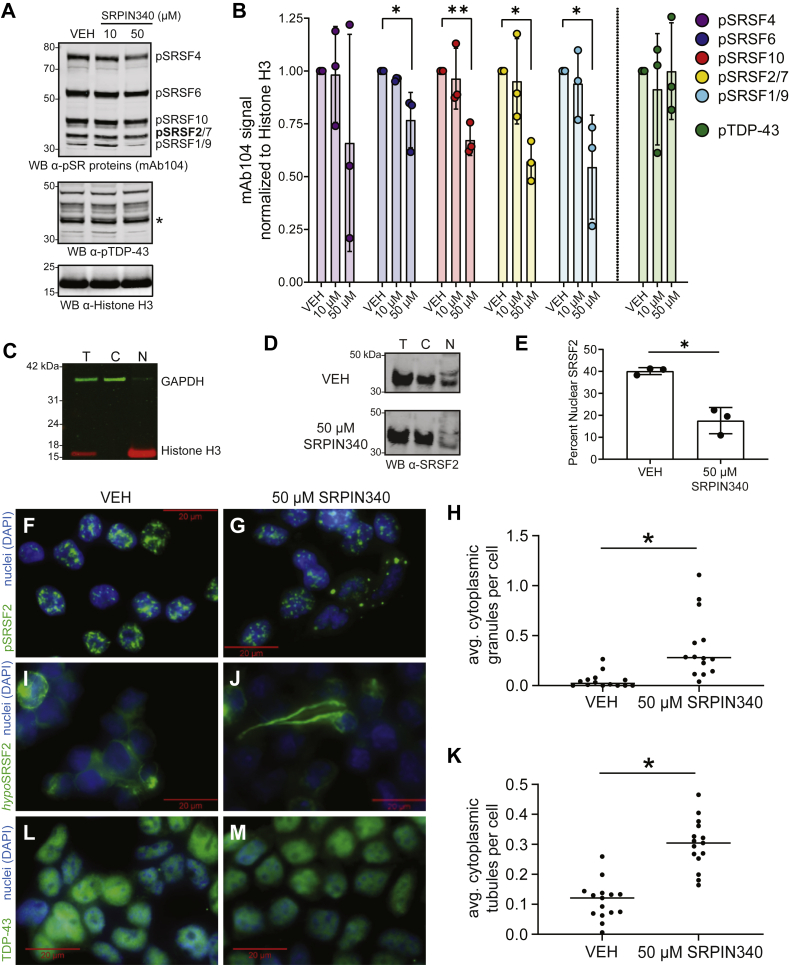
**Inhibiting SRPKs decreases SR protein phosphorylation and increases cells harboring cytoplasmic SRSF2 granule and tubule structures in HEK293 cells.***A*, HEK293 cells were incubated with either DMSO (vehicle, VEH) or increasing concentrations of SRPK inhibitor SRPIN340 for a length of 12 h. Immediately following treatment, cells were harvested, run by SDS-PAGE, and immunoblotted for phosphoSR (pSR) proteins using the pan-pSR antibody mAb104. A p-TDP-43 antibody (pTDP-43 band marked by *asterisk*) and a Histone H3 antibody were used to confirm specificity of SRPIN340 and equal protein loading, respectively. *B*, protein band intensities for SRSF4 (*purple*), SRSF6 (*blue*), SRSF10 (*red*), SRSF2/SRSF7 (*gold*), SRSF1/9 (*turquoise*), and separately, pTDP-43 (*green*) were quantified and normalized to the VEH condition (artificially set to value = 1) (multiple *t* tests, ∗*p* < 0.05, ∗∗*p* < 0.01, ∗∗∗*p* < 0.001). Error bars indicate maximum and minimum ranges of band values. The pSRSF2 signal was significantly decreased at 50 μM SRPIN340 concentration, whereas fellow RBP TDP-43 was not. *C*, validation of HEK293 cell extract fractionation procedure, yielding total (T), cytosolic (C), and nuclear (N) samples. The fractions were immunoblotted for the cytosolic marker GAPDH (*green*) and nuclear marker Histone H3 (*red*). *D*, immunoblot of nuclear-cytoplasmic fractions for total, endogenous SRSF2 in VEH and SRPIN340 conditions. *E*, quantification of percent nuclear SRSF2 (nuclear signal divided by sum of cytoplasmic and nuclear signals) in VEH and SRPIN340 conditions (paired *t* test, ∗*p* value = 0.0130). *F* and *G*, immunocytochemical (ICC) staining of HEK293 cells for phosphoSRSF2 (pSRSF2)-positive nuclear speckles (*green*) and DAPI+ nuclei (*blue*) in vehicle-treated cells (*F*) and 50 μM SRPIN340-treated cells (*G*). *H*, the number of cytoplasmic granules observed was divided by the number of cells counted and averaged for 14 independent images in three independent replicates (two-tailed paired *t* test, ∗*p* value = 0.0191). A minimum of 120 cells were counted in each condition per replicate. The error bars represent the range of standard deviation. *I* and *J*, ICC staining of HEK293 cells for hypophosphorylated SRSF2 (hypoSRSF2) (*green*) and DAPI+ nuclei (*blue*) in vehicle-treated cells (*I*) and 50 μM SRPIN340-treated cells (*J*). *K*, the fraction of cells harboring cytoplasmic SRSF2 tubule structures was quantified in four biological replicates and compared (two-tailed paired *t* test, ∗*p* value = 0.0178). *L*, vehicle- and (*M*) 50 μM SRPIN340-treated HEK293 cells were stained by ICC for TDP-43 (*green*) and DAPI-stained.

Having established that SRSF2 aggregates and forms high-molecular-weight oligomers upon dephosphorylation *in vitro*, we attempted to inhibit SR protein phosphorylation in cell culture. We incubated HEK293 cells in media containing the compound SRPIN340. To validate SRPIN340 we analyzed cell lysates by western blot using mAb104 (63), an antibody that labels phospho epitopes on numerous SR proteins (64) (Fig. 5*A*). Quantification of mAb104 immunoblot signal intensities normalized to Histone H3 demonstrates that several SR proteins, particularly SRSF2, exhibit decreased phosphorylation with increasing concentrations of SRPIN340 (Fig. 5*B*). Independently, we compared the pSR protein labeling with that of phosphoTDP-43 (pTDP-43) (26). We found that while pTDP-43 did not quantitatively change with SRPIN340 treatment, pSRSF2 signal was significantly decreased at 50 μM SRPIN340. These results demonstrate that inhibition of SRPKs using SRPIN340 successfully reduces pSRSF2 levels, as well as overall SR protein phosphorylation.

Next, we asked whether SR protein phosphorylation dysregulation could fundamentally alter the localization of the RBP SRSF2. Using a nuclear-cytoplasmic fractionation procedure (Fig. 5*C*) (28, 65), we isolated cytoplasmic and nuclear fractions from cells that underwent either vehicle or SRPIN340 treatment (Fig. 5*D*). In HEK293 cells, SRSF2 appeared to enrich in cytoplasmic and nuclear extracts at similar levels. However, in SRPIN340-treated cells, SRSF2 was primarily cytoplasmic. Quantification of nuclear fraction abundances (percent nuclear SRSF2) demonstrates that SRSF2 is significantly less nuclear in SRPIN340-treated cells (∼18%) *versus* vehicle-treated cells (∼40%) (Fig. 5*E*). Therefore, we demonstrate that SRSF2 is enriched in cytoplasmic fractions when its phosphorylation state is decreased. The cytoplasmic mislocalization of SRSF2 following pharmacological inhibition of SRPKs was also demonstrated with an antibody raised against a nonphospho epitope of SRSF2 (Fig. S5).

As we observed high-molecular-weight species of dephosphorylated SRSF2 *in vitro*, we hypothesized that inhibiting SR protein kinases within cells would induce an increase in cytoplasmic granules and/or the formation of fibril-like species, hallmarks of various RBP proteinopathies (66). Immunocytochemical (ICC) staining using the nuclear speckle antibody SC35 demonstrated canonical nuclear speckle morphology of pSRSF2 in vehicle-treated cells (Fig. 5*F*). In cells incubated with SRPIN340, however, we observed unusual cytoplasmic granules (Fig. 5*G*). We performed quantification and observed a significant increase in the average number of cytoplasmic granules per cell in the SRPIN340 condition (Fig. 5*H*). We next labeled cells using the *hypo*SRSF2 antibody, observing dephosphorylated SRSF2 dispersed largely throughout the cytoplasm, and infrequently observed as small tubules extending from the cell body outward (Fig. 5*I*). In cells incubated with SRPIN340, however, we observed a significant increase in cells harboring cytoplasmic SRSF2 tubule structures, which were substantially larger in size (Fig. 5, *J* and *K*). As expected, granule condensation and formation of tubule-like filaments were not observed for TDP-43, which showed a consistent pattern of nuclear distribution in both conditions (Fig. 5, *L* and *M*).

### SRSF2 interacts with microtubule subunit proteins α- and β-tubulin

**Figure 6:**
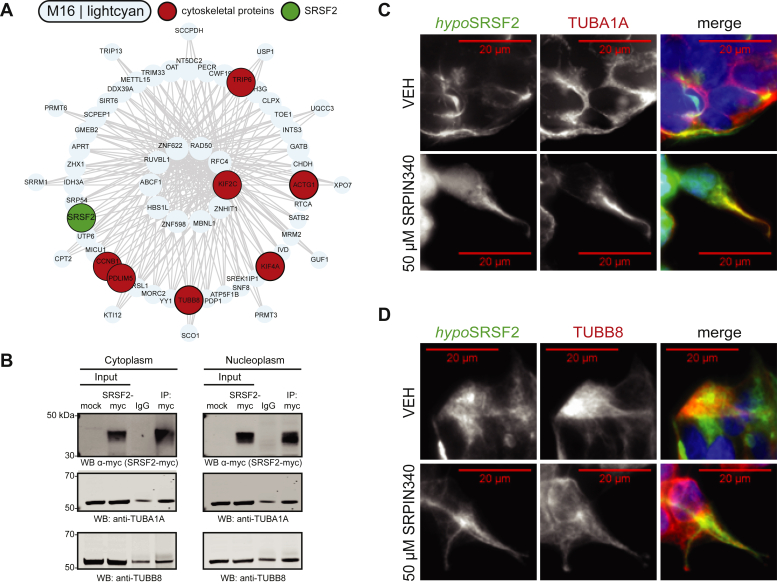
**Association of SRSF2 with microtubule proteins.***A*, I-graph of module 16 (M16) representing hub proteins and corresponding gene symbols as nodes. Node size and edges (*gray*) are reflective of the degree of intramodular connectivity in WGCNA. SRSF2 (*green*) and cytoskeletal-associated proteins (*red*) are highlighted. *B*, representative western blot of an immunoprecipitation (IP) of recombinant SRSF2-myc in cytoplasm and nucleoplasm extracts isolated from HEK293 cells (*n* = 3 replicates per fraction). Co-IP complexes were blotted for α-tubulin (TUBA1A) and β-tubulin (TUBB8). *C* and *D*, ICC staining of HEK293 cells for hypophosphorylated SRSF2 (hypoSRSF2) (*green*) and both TUBA1A (*red*; *C*) and TUBB8 (*red*; *D*) in vehicle-cells or 50 μM SRPIN340-treated cells.

Given the unusual tubular morphology we observed of hypophosphorylated SRSF2 in cells and enhanced aggregation of SRSF2 following dephosphorylation *in vitro,* we further examined constituents of module M16, of which SRSF2 is a member (Fig. 6*A*). Interestingly, several proteins within this module are components of the cytoskeleton, including microtubule subunit protein β-tubulin 8 (TUBB8). To answer whether SRSF2 interacts with microtubules, we immunopurified recombinant SRSF2 and immunoblotted for TUBB8 and TUBA1A. We indeed found that SRSF2 interacts with TUBB8 and TUBA1A (Fig. 6*B*). Furthermore, ICC staining of SRSF2 with either TUBA1A or TUBB8 demonstrates that dephosphorylated SRSF2 preferentially associates with microtubule structures (Fig. 6, *C* and *D*). Thus, network-based proteomics revealed an association between arginine-rich RBPs such as SRSF2 and microtubules, supported by both biochemical and cell imaging approaches.

## Discussion

**Figure 7:**
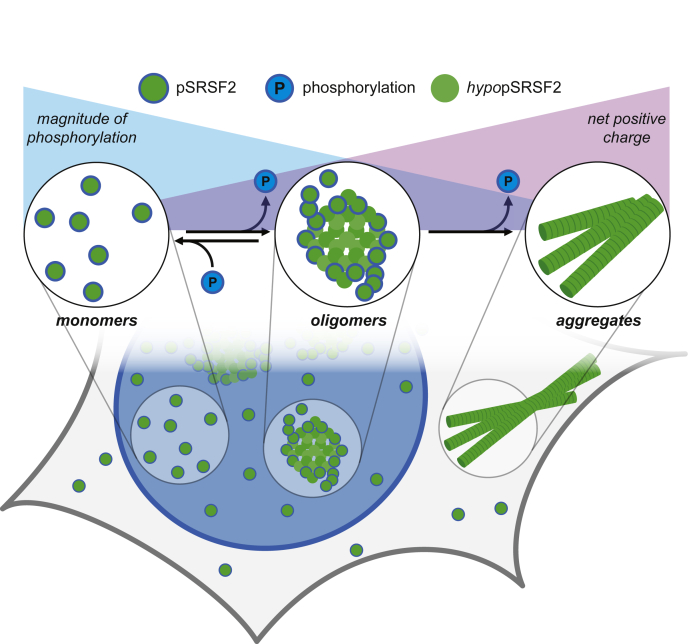
**Proposed model of SRSF2 solubility and oligomerization changes regulated by phosphorylation.** Equilibrium of structural states (monomer, oligomer, aggregate) of SRSF2 (*green*) in varying states of phosphorylation (*dark turquoise border*). The magnitude of positive (*purple triangle*) SRSF2 net charge is illustrated, inverse to the degree of phosphorylation (*turquoise triangle*). Phosphorylation regulates higher-order SRSF2 solubility, oligomerization, and structure formation.

Here we used a mass-spectrometry-based proteomics approach to investigate how phosphorylation affects protein solubility and oligomerization. Using systems biology analyses, we identified modules of proteins with shared biology and sequence homology that coaggregate following dephosphorylation. Arginine-rich proteins, including SRSF2, were among the proteins with the most decreased solubility following dephosphorylation. SRSF2 was used as a paradigm to investigate the relationship between phosphorylation and protein structure and solubility. We discovered that dephosphorylation regulates higher-order multimer formation of SRSF2, whereas phosphorylated SRSF2 more frequently exists as a monomer species *in vitro.* Inhibition of SR protein kinases within mammalian cells decreased SR protein phosphorylation, resulting in increased cytoplasmic SRSF2 localization and granule formation. We also observed hypo-phosphorylated SRSF2 to form unusual cytoplasmic tubule structures, which colocalize with cytoskeletal proteins. Collectively, these data suggest that phosphorylation tunes SRSF2 net charge, solubility, and structure by virtue of multimer disassembly *in vitro* and *in vivo* (Fig. 7).

SRSF2 is a well-studied classical SR protein with integral roles in constitutive and alternative splicing (30). This study highlights numerous functions of SRSF2 that are regulated by phosphorylation and are associated with RBP dysregulation in neurodegenerative disease, including decreased solubility, high-molecular-weight oligomer formation, and cytoplasmic mislocalization and granule and tubule-like morphology formation. Another phenotype of dysregulated RBPs in neurodegenerative disease is the accumulation of splicing errors (67, 68, 69) which could possibly be influenced by SR protein phosphorylation status. Given the substantial solubility and localization changes of SRSF2 we observed upon SRPK inhibition using SRPIN340, we hypothesize that many genes would be alternatively spliced as a result of SR protein hypophosphorylation. Use of the broad SRPK inhibitor SRPIN340 may provide a stronger effect of SR protein phosphorylation suppression than siRNA-mediated knockdown alone (53). Indeed, several groups have discovered altered splicing as a result of SRPK inhibition using SRPIN340 in mammalian cells (70, 71). Future studies investigating the regulation of the SR protein splicing network by phosphorylation may help to resolve specific genes susceptible to splicing defects upon kinase dysregulation in disease.

Although mutations in SRSF2 are frequently observed in individuals with myelodysplastic syndromes (MDS) or chronic myelomonocytic leukemia (CMML) (72, 73, 74, 75, 76), SRSF2 has not been commonly associated with human neurodegenerative disease. Recently, however, the McKnight group demonstrated that SRSF2 does indeed form condensates *in vitro*, a hallmark feature of RBPs that aggregate in neurodegenerative disease, which importantly was reversible by phosphorylation (77, 78). While it is generally considered that phosphorylation promotes protein aggregation, a notable finding of this study is that arginine-rich RBPs, such as the SR and LUC7L protein families, exhibit remarkably similar aggregation patterns when dephosphorylated. Indeed, our group and others have observed that arginine-rich splicing proteins mislocalize and aggregate in AD brain (14, 15, 79, 80). As kinases are dysregulated in AD (25), SRSF2 is a protein susceptible to solubility dysregulation in disease. Remarkably, SRSF2 was recently identified as a novel protein that associates with phosphorylated tau in AD brain by proteomic examination of microdissected neurofibrillary tangles (81). Our group has not identified SRSF2 as a protein with significantly increased insolubility in AD (82). However, our studies have been limited to the sarkosyl-insoluble fractions only. Future studies that include internal comparisons of insoluble to soluble fractions may implicate SRSF2 and similar RBPs that are depleted from AD brain homogenate soluble fractions and may reveal novel proteins that undergo altered solubility in AD.

We highlight phosphorylation as a critical feature of RBPs that strongly influences protein solubility. An important consideration is the influence that similarly negatively charged RNA molecules may hold over the solubility of nuclear proteins. Future investigations that explore the role of RNA in regulating the solubility of RBPs may yield a parallel interpretation of RBP stability. Further still, studies that investigate the role of phosphorylation in the regulation of arginine-rich RBP aggregation may hold promise to reveal the mechanism underlying RBP aggregation in neurodegenerative diseases.

## Experimental procedures

### Materials

The primary antibodies used in this study include: rabbit polyclonal anti-myc (Cell Signaling Technology 2272S), rabbit polyclonal anti-SRSF2 (Abcam ab229473), mouse monoclonal anti-SRSF2 (Clone 1Sc-4F11, Millipore Sigma 04-1550), mouse monoclonal anti-pSRSF2 (Abcam ab11826), rabbit polyclonal anti-LUC7L (Thermo Fisher 17085-1-AP), rabbit polyclonal anti-LUC7L3 (Thermo Fisher PA5-53816), rabbit polyclonal anti-RBM25 (Abcam ab72237), rabbit polyclonal anti-SRSF1 (Abcam ab38017), rabbit polyclonal anti-TDP-43 (ProteinTech Group 10782-2-AP), rabbit anti-pS409-410 TDP-43 (CosmoBio, CAC-TIP-PTD-P02), rabbit polyclonal anti-panQKI (generated by UC Davis/NIH NeuroMab Ab_10671658), rabbit polyclonal anti-ZC3H18 (Thermo Fisher PA5-59322), rabbit polyclonal anti-SRRM1 (ab221061), mouse anti-GAPDH (Abcam ab8245), rabbit polyclonal anti-Histone H3 (Abcam ab1791), rat monoclonal anti-alpha-tubulin clone YL1/2 (MAB1864), rabbit polyclonal anti-TUBB8 (ab97880), and mouse IgM anti-pSR proteins (Clone mAb104, ATCC CRL-2067, see below isolation method). Each antibody save for mAb104 was used at a 1:1000 dilution in blocking buffer for western blotting. Each antibody used for ICC was diluted 1:500 in normal horse serum (NHS). The pcDNA3.1-SC35-cMyc SRSF2 plasmid was a gift from Kathleen Scotto (83) (Addgene plasmid #44721).

### mAb104 isolation

Immortalized mouse hybridomas were purchased from ATCC (CRL-2067). Cells were thawed and then passaged twice before collection. Forty-eight hours postpassaging, the cell suspension media was aliquoted into a 15 ml conical tube, then spun at 250*g* for 5 min. The supernatant was aspirated, and cells were resuspended in 2%FBS in DMEM. Cells were cultured for 3 more days, then the cell suspension was aliquoted into a 15 ml conical tube, then spun at 1500*g* for 15 min. The supernatant was transferred to a fresh 15 ml conical tube, then aliquoted into microcentrifuge tubes. The supernatant aliquot volumes were then reduced under SpeedVac (Labconco 731022) to 50% of original volume and restored to the original volume with 100% glycerol. Tubes were then frozen at −20C. The antibody was diluted 1:5 in blocking buffer for western blotting.

### Local charge density and NCPR

Residue charge at physiological pH (7.4) was calculated using a simple algorithm modeled from EMBOSS. The residues D/E were counted with charge = −1, residues K/R with charge = +1, and H with charge = +0.5. Residues S/T/Y were counted as either charge = 0 or −2, to mimic the charge assumed after phosphorylation posttranslational modification at physiological pH (56). To generate the local charge density, the average charge over a window of 21 residues starting at the C-terminus was calculated, sliding toward the N-terminus of the protein. To calculate the Net Charge per Residue (NCPR) of full proteins, the residue charges, as calculated above, were summed, and this number was divided by the number of residues in the protein. The NCPR of the average protein of the known human proteome is ∼ +0.013, spanning from −0.40 to 0.32 (minimum protein size = 100 residues).

### Cell culture and transfection

HEK293 cells were cultured in Dulbecco’s Modified Eagle Medium [DMEM, high glucose (Gibco)] supplemented with 10% (v/v) fetal bovine serum (Gibco) and 1% penicillin-streptomycin (Gibco) and maintained at 37 °C under a humidified atmosphere of 5% (v/v) CO_2_ in air. Cells were grown to 70 to 80% confluency in 10 cm^2^ culture dishes and transfected with 10 μg SRSF2-myc plasmid and 30 μg linear polyethylenimine (PEI). Cells were harvested and fractionated to enrich for nucleoplasm as described below. For western blotting SRPIN340-inhibitor treated cells, HEK293 cells were incubated for 12 h (84) with equivolume amounts of either DMSO (vehicle) or SRPIN340 (final concentration = 50 μM) starting 24 h post-passage. Afterward, cells were harvested in IP lysis buffer (50 mM HEPES pH7.4, 150 mM NaCl, 5% Glycerol, 1 mM EDTA, 0.5 (v/v) NP-40, 0.5% (v/v) CHAPS) and sonicated at 25% amplitude for 3 × 10 s on/off cycles. Lysates were then cleared after a 15,600*g* centrifugation step at 4 °C. Supernatants were transferred to new tubes and run by SDS-PAGE followed by western blotting.

### Nucleoplasm enrichment

This cellular extraction procedure (65) was modified to include NP-40 detergent. In short, 48 h posttransfection, HEK293 cells were rinsed with cold PBS then scraped in PBS+1xHALT protease/phosphatase inhibitor (Thermo Fisher). The cell slurry was centrifuged for 5 min at 1000*g* at 4 °C to pellet cells. The supernatant was aspirated, and the cells were washed with 1 ml PBS+1xHALT (100 μl taken as total fraction) and centrifuged again to pellet cells. The supernatant was aspirated, and the cells were swelled in 150 μl Hypotonic Lysis Buffer+1xHALT (10 mM HEPES pH 7.9, 20 mM KCl, 0.1 mM EDTA, 1 mM dithiothreitol (DTT), 5% Glycerol, 0.5 mM PMSF, 10 μg/ml Aprotinin, 10 μg/ml Leupeptin, 0.1% NP-40) and incubated on ice for 5 min. The sample was then centrifuged for 10 min at 15,600*g* at 4 °C. The supernatant was collected as the cytoplasmic fraction, and the resulting pellet was incubated in 100 μl High Salt Buffer+1xHALT (20 mM HEPES pH 7.9, 0.4 M NaCl, 1 mM EDTA, 1 mM EGTA, 1 mM DTT, 0.5 mM PMSF, 10 μg/ml Aprotinin, 10 μg/ml Leupeptin) for 30 min on ice to extract nuclei. The samples were then sonicated for 5 s at 25% amplitude and centrifuged at 18,213*g* for 10 min at 4 °C. The supernatant was collected as the nucleoplasm fraction, and the pellet (chromatin fraction) was resuspended and sonicated in Nuclei lysis buffer (50 mM Tris-HCl pH 8.0, 10 mM EDTA, 1% SDS). All fractions were frozen at −70 °C, and only the cytoplasm and nucleoplasm fraction were used for following applications.

### Calf intestinal phosphatase (CIP) treatment

For dephosphorylation assays, nucleoplasm fractions (100 μg) were incubated with either mock (distilled water) or 50 Units of calf intestinal alkaline phosphatase (QuickCIP, NEB M0525L) for 1 h at 37 °C. Following this, Laemmli sample buffer (8% glycerol, 2% SDS, 50 mM Tris pH 6.8, 3.25% beta-mercaptoethanol) was added to each sample to 1× and boiled at 95 °C for 10 min and run by SDS PAGE. For sedimentation assays, nucleoplasm fractions (100 μg) were incubated with either distilled water (mock) or 50 Units of CIP (QuickCIP, NEB M0525L) and brought up to 100 μl with PBS. The samples were heated at 37 °C for 1 h.

### Sedimentation assay

Following QuickCIP dephosphorylation, 50 μl of the 100 μl sample was added to polycarbonate ultracentrifuge tubes. Samples were spun at 100,000*g* for 1 h at 4 °C. The supernatant (vol = 50 μl) was transferred to a LoBind tube (Eppendorf 0030108442), and the insoluble pellet was resuspended in 50 μl 8M Urea and transferred to a separate LoBind tube. The pellet sample was sonicated for 1 s on/off cycles at 25% amplitude until the pellet disappeared. For western blotting, 7.5 μl of total (prespin) fractions was added and 15 μl of soluble and pellet fractions were added. For mass spectrometry analysis, 30 μl of soluble and pellet fractions was used.

### Western blotting

Western Blotting was performed according to standard protocol as previously described (28). In short, samples were boiled in Laemmli sample buffer (8% glycerol, 2% SDS, 50 mM Tris pH 6.8, 3.25% beta-mercaptoethanol) for 10 min, then resolved on a Bolt 4 to 12% Bis-tris gel (Invitrogen NW04120BOX) by SDS-PAGE, and semidry transferred to a nitrocellulose membrane with the iBlot2 system (Thermo Fisher IB21001). Membranes were blocked with TBS Starting Block Blocking Buffer (ThermoFisher 37542) and probed with primary antibodies (1:1000 dilutions) overnight at 4 °C. Membranes were then incubated with secondary antibodies conjugated to either Alexa Fluor 680 or 800 (Invitrogen) fluorophores for 1 h at RT. Membranes were imaged using an Odyssey Infrared Imaging System (Li-Cor Biosciences), and band intensities were calculated using Odyssey imaging software.

### Sample preparation for mass spectrometry analyses

Thirty microliters of soluble and pellet fractions from four separate biological replicates was normalized to 50 μl with 8M urea buffer. Next, 10 mM Dithiothreitol (DTT) in 50 mM ammonium bicarbonate (ABC) was added to a final concentration of 1 mM and incubated for 30 min at room temperature (RT), then 50 mM iodoacetamide (IAA) in 50 mM ABC was added to a final concentration of 5 mM and incubated in the dark for 30 min at RT. Samples were then digested overnight with 1 μg of Lys-C (Wako 121-05063). Following Lys-C digestion, samples were diluted to 1M urea and digested overnight with 1 μg of Trypsin (Thermo Fisher 90057). The next day samples were incubated with acidifying buffer [10% Formic Acid (FA), 1% trifluoroacetic acid (TFA)] and centrifuged for 2 min. Sample pH was verified as less than 3 using pH strips. Samples were desalted on an Oasis PRIME HLB 10 mg plate (Oasis 186008053) and washes were flowed through columns by a 96-well Positive Pressure processor (Waters 186006961). Samples were washed first with methanol, then Buffer A (0.1% TFA). The digested samples were then loaded onto Oasis PRIME HLB 10 mg plates (Oasis 186008053), washed with Buffer A twice, and then peptides were eluted with Buffer C [50% acetonitrile (ACN), 0.1% FA]. The elutant was lyophilized using a SpeedVac (Labconco 731022).

### Mass spectrometry analysis

Lyophilized peptides were resuspended in loading buffer (0.1% FA, 0.03% TFA, 1% ACN) and separated on a self-packed C18 (1.9 μm Dr Maisch, Germany) fused silica column (20 cm × 75 μm internal diameter; New Objective) by a NanoAcquity UHPLC (Waters). Linear gradient elution was performed using Buffer A (0.1% formic acid, 0% acetonitrile) and Buffer B (0.1% formic acid, 80% acetonitrile) starting from 3% Buffer B to 40% over 100 min at a flow rate of 300 nl/min. Mass spectrometry was performed on an Orbitrap Fusion Lumos Mass Spectrometer in top speed mode. One full MS1 scan was collected followed by as many data-dependent MS/MS scans that could fit within a 3 s cycle. MS1 scans (400–1600 m/z range, 400,000 AGC, 50 ms maximum ion time) were collected in the Orbitrap at a resolution of 60,000 in profile mode with FAIMS CV set at −45. The MS/MS spectra (1.6 m/z isolation width, 35% collision energy, 10,000 AGC) were acquired in the ion trap. Dynamic exclusion was set to exclude previous sequenced precursor ions for 30 s with a mass tolerance of 10 ppm.

### Database searching and label-free quantification

Data files for the 16 samples were analyzed using MaxQuant v1.6.17.0 with Thermo Foundation 2.0 for RAW file reading capability. The search engine Andromeda (85) was used to build and search a concatenated target-decoy UniProt Knowledgebase (UniProtKB) containing both Swiss-Prot and TrEMBL human protein sequences (86,395 sequences, downloaded August 9, 2020) with 245 contaminant proteins as a parameter for the search (86). Methionine oxidation (+15.9949 Da), protein N-terminal acetylation (+42.0106 Da), and STY-phosphorylation (+79.966 Da) were included as variable modifications (up to five allowed per peptide); cysteine was assigned a fixed carbamidomethyl modification (+57.0215 Da). Fully tryptic peptides were considered with up to two miscleavages allowed in the search. A precursor mass tolerance of ±20 ppm was applied prior to mass accuracy calibration and ±4.5 ppm after internal MaxQuant calibration. The false discovery rate (FDR) for peptide spectral matches, proteins, and site decoy fraction were all set to 1%. Phosphorylated peptides were not quantified. Protein group intensity calculated by the label-free quantitation (LFQ) algorithm in MaxQuant (87, 88) was used for protein quantitation. The quantitation method did not consider reverse, contaminant, and by site only protein identifications, leaving 5017 proteins for downstream analysis.

### Weighted gene correlation network analysis of nuclear fractionation proteome

Prior to network analysis, the nucleoplasm proteome (5017 proteins) was culled to select for proteins with less than or equal to 50% missing values (intensity values in eight out of 16 samples; 4366 proteins). Protein groups with a single peptide identification were removed, leaving 4120 proteins. The intensity values were then log_2_-transformed. Missing protein intensity values were imputed using random numbers drawn from a normal distribution of observed protein intensity values of each sample (width = 0.3, down shift = 1.8, calculated separately for each column) in Perseus v1.6.15.0. The R package WGCNA v1.68 was used to cluster proteins by abundance into groups of proteins with similar solubility patterns using a dissimilarity metric for clustering distance based on 1 minus the topology overlap matrix (1-TOM), a calculation based on an adjacency matrix of correlations of all pairs of proteins in the abundance matrix supplied to WGCNA (57). The weighted protein coexpression network was built using the log_2_-transformed intensity values using the blockwiseModules function with the following parameters: soft threshold power beta = 20, deepSplit = 4, minimum module size = 15, merge cut height = 0.07, signed network partitioning about medioids respecting the dendrogram, and a reassignment threshold of *p* = 0.05. GO Elite v1.2.5 python package was used as previously published to categorize summary biological functions of individual modules (89, 90). Z scores were determined by one-tailed Fisher’s exact text (Benjamini–Hochberg FDR corrected) to demonstrate overrepresentation of ontologies in the nucleoplasm proteome of each module. The filters included a cutoff for Z scores as 1.96, *p* value cutoff of 0.01 and a minimum of five genes per ontology.

### Differential solubility analysis

Proteins either differentially soluble or differentially insoluble following dephosphorylation were calculated using a two-tailed paired *t* test on the fraction insolubility values according to a previous study by our group (91):

$${\log\;}_{2}{\left[\text{insoluble}/\left(\text{insoluble }+\text{ soluble}\right)\right]}_{+\text{CIP}}\;-\;{\log\;}_{2}{\left[\text{insoluble}/\left(\text{insoluble }+\text{ soluble}\right)\right]}_{\text{mock}}$$

Proteins were highlighted as enriched/depleted from a fraction if greater than or equal to a twofold change in fraction insoluble values (±log_2_ (2)=1) was observed with a *p*-Value less than 0.05 (two-tailed paired *t* test).

### SR protein similarity and insolubility enrichment assessment

The RS domain (AA117-221) of SRSF2 was searched September 15, 2021 for similarity using the Uniprot pBLAST feature (http://www.uniprot.org/blast/) using the following parameters: Target Database Human, E-threshold: 10, Matrix: Auto, Filtering: None, Gapped: Yes, Hits: 1000. Similar proteins were then filtered to remove unreviewed protein entries, leaving 193 proteins with E-values less than 0.005 and similarity to the SRSF2 RS domain of greater than 20% (Table S6). Protein alignment was performed using Clustal Omega multiple sequence alignment (https://www.ebi.ac.uk/Tools/msa/clustalo/). The list of 193 proteins was then compared with the list of proteins with increased insolubility following dephosphorylation (*n* = 734) using a hypergeometric Fisher’s exact test overlap using R.

### SRPK2 *in vitro* kinase reaction

Human recombinant SRPK2 (0.4 −μg/reaction; EMD Millipore 14-666; Glu46-end) and SRSF2 (0.8 μg/reaction; MyBioSource, Inc MBS2029592; Thr14-end) expressed in *E. coli* were purchased and added to Kinase Buffer I (25 mM MOPS pH7.2, 12.5 mM β-glycerol phosphate, 25 mM MgCl_2_, 5 mM EGTA, 2 mM EDTA, 0.25 mM DTT; Abcam ab189135). Different concentrations (10, 50, 100 μM) of SRPK inhibitor SRPIN340 were added prior to SRSF2 substrate. Either water (mock) or ATP was added to a final concentration of 100 μM to a final volume of 25 μl and incubated for 30 min at 30 °C. Reactions were then separated by denaturing or nondenaturing Blue native Gel Electrophoresis or by Isoelectric focusing (IEF).

Radioactive kinase assays were performed as previously described (92). In short, kinase reactions were set up in Kinase Buffer I with 1 μM “cold” ATP and 10 μCi γ–[^32^P]ATP or only 10 μCi γ–[^32^P]ATP totaling 10 μl. Reactions were incubated for 10 min at 30 °C and stopped by the addition of SDS loading buffer and analyzed by both SDS-PAGE and autoradiography.

### Blue native gel electrophoresis

Blue native PAGE was carried out as described previously (16). Purified recombinant SRPK2 and SRSF2 proteins (0.4 and 0.8 μg, respectively) were incubated with blue native gel loading buffer [5% glycerol, 50 mM TCEP (Sigma-Aldrich 646547), 0.02% (w/v) Coomassie G-250 (Invitrogen BN2004), 1× NativePAGE Sample Buffer (Invitrogen BN2003)] in LoBind tubes (Eppendorf 0030108442) for 30 min at room temperature. Mock and CIP-treated nucleoplasm lysate (50 μg) were incubated with the same blue native gel loading buffer and incubated on ice for 30 min. Samples and Blue Native Protein Ladder (ThermoFisher LC0725) were loaded onto NativePAGE Bis-Tris Gels (3–12%, Invitrogen BN1001BOX), and gel electrophoresis was performed using anode NativePAGE Running Buffer (Invitrogen BN2001) and cathode buffer (Invitrogen BN2002) with additive (Invitrogen BN2004) and run for 15 min at 150V. The Dark Blue Cathode Buffer was then interchanged with Light Blue Cathode Buffer, proceeding for another 90 min at 150 V. Following this, the gel was gently rocked in 50 mM Tris-HCl, pH 7.5, and 1% SDS for 30 min and transferred using the semidry iBolt transfer system (Invitrogen) with PVDF membrane (Invitrogen IB24002) for 7 min at 20 V. Following transfer, the PVDF membrane was rocked in 8% acetic acid solution for 5 min, rinsed with distilled water, air dried, then rinsed with methanol. The membrane was then blocked, western blotted, and imaged on the Odyssey Infrared Imaging System (Li-Cor Biosciences).

### Immunocytochemistry

Immunocytochemical staining was carried out as previously described (93). HEK293 cells were grown on Nunc Lab-Tek II Chamber Slide systems (ThermoFisher 154534PK). Twenty-four hours postpassage, HEK293 cells were incubated with equivolume amounts of either DMSO (vehicle) or SRPIN340 (final concentration = 50 μM). Cells were incubated at 37 °C under a humidified atmosphere of 5% (v/v) CO_2_ in air for 4 h. Cell media was aspirated then washed 3× warm sterile PBS for 5 min each. Cells were then fixed with 4% paraformaldehyde (Electron Microscopy Sciences 15713S) diluted in PBS for 45 min at RT and afterward washed 3× warm sterile PBS for 5 min each. Cells were then incubated with 0.05% Triton X-100 diluted in PBS for 20 min at RT. Afterward, cells were blocked with 10% normal horse serum (NHS) in PBS for 45 min at RT. The blocking solution was aspirated and excess liquid was removed with a kimwipe. The cells were then incubated in primary antibody diluted in 2% NHS/1xPBS overnight at 4 °C. The next day, cells were washed 3× warm sterile PBS for 5 min each. Cells were then incubated in secondary antibody diluted in 2% NHS/1xPBS for 1 h at RT then washed 3× warm sterile PBS for 5 min each. PBS was aspirated and a droplet of DAPI-containing mounting media (Abcam ab104139) was placed on cells, which were coverslipped and sealed with clear nail polish. Images were captured on a Keyence BZ-X810 widefield laser scanning microscope (Keyence). At least 14 images were taken for each condition per biological replicate at random areas of the slide.

### Imaging quantification and statistical analysis

Images were analyzed with FIJI. Graphs were developed with GraphPad Prism. Over 100 cells each were counted (DAPI+ nuclei) and analyzed per three biological replicates. Then, the number of SC35+ (pSRSF2) granules was counted in each image and divided by total cells observed. The average number of granules per cell per image (*n* = 14) was collected for each biological replicate. At least 120 cells were counted in each condition per replicate. The number of cytoplasmic granules per cell was averaged for each replicate and conditions were statistically compared using a two-tailed paired *t* test. To measure the percent of cells with cytoplasmic SRSF2 tubules, the total number of DAPI+ nuclei was counted as the number of cells per image. A total of 15 images per biological replicate were recorded, with four biological replicates analyzed. Then, the number of cells that contained an SRSF2 tubule morphology was counted. The number of SRSF2 tubule-containing cells was then divided by the number of DAPI+ nuclei and expressed as a decimal. The number of SRSF2 tubules per cell was averaged in each replicate and the vehicle and drug conditions were statistically compared using a two-tailed paired *t* test.

### Immunoprecipitation

Immunoprecipitations were performed essentially as described (28). Briefly, HEK-293 cells were transiently transfected with either myc-tagged SRSF2 plasmid or a mock control vector. Harvested cells were fractionated to isolate the nucleoplasm and cytoplasm as described above. Protein concentrations were determined using a bicinchoninic acid (BCA) assay (Thermo Fisher 23225). Approximately 20 μl of Protein A Sepharose 4B beads (catalog no. 101042, Invitrogen) was washed twice in immunoprecipitation (IP) buffer [50 mM HEPES pH 7.4, 150 mM NaCl, 5% glycerol, 1 mM EDTA, 0.5% (v/v) NP-40, 0.5% (v/v) CHAPS, HALT phosphatase inhibitor cocktail (1:100, Thermo Fisher 87786)], then blocked in 0.1 mg/ml bovine serum albumin (Thermo Fisher 23209), then washed an additional three times in IP buffer. Anti-myc (4 μg) mouse monoclonal antibody (Cell Signaling 2276) or mouse isotype IgG control (4 μg, BD Pharmingen 550339) was incubated with the bead slurry in IP buffer and rotated for 90 min to conjugate antibody to beads. The beads were then washed three times in IP buffer, then nucleoplasm lysates were added to beads (0.2 mg per IP) and incubated rotating overnight at 4 °C. The beads were then washed three times in IP wash buffer (IP buffer lacking glycerol and CHAPS), then resuspended in IP wash buffer. Preceding the final wash, the bead slurry was transferred to a new microcentrifuge tube to limit contamination. The bead slurry was then centrifuged at 500*g* for 5 min at 4 °C, and the pelleted beads were resuspended with 1× Laemmli sample buffer (8% glycerol, 2% SDS, 50 mM Tris pH 6.8, 3.25% β-mercaptoethanol) and run by SDS-PAGE. Nucleoplasm fractions (10 μg) were included as input loading controls. Three independent biological replicates were performed, and a representative blot image was shown.

## Data availability

The mass spectrometry proteomics data have been deposited to the ProteomeXchange Consortium *via* the PRIDE partner repository (94) with the dataset identifier PXD026894 at http://proteomecentral.proteomexchange.org/cgi/GetDataset?ID=PXD026894 on June 23, 2021.

## Supporting information

This article contains supporting information.

## Conflict of interest

The authors declare that they have no conflicts of interest with the contents of this article.
