# Supplementary material for: Phosphorylation regulates arginine-rich RNA-binding protein solubility and oligomerization
Source: J Biol Chem. 2021 Oct 19;297(5):101306. doi: 10.1016/j.jbc.2021.101306 (PMC8569591; doi:10.1016/j.jbc.2021.101306)
Supplement: Figures S1–S5 [file mmc2.pdf]

Supplemental Figure S1

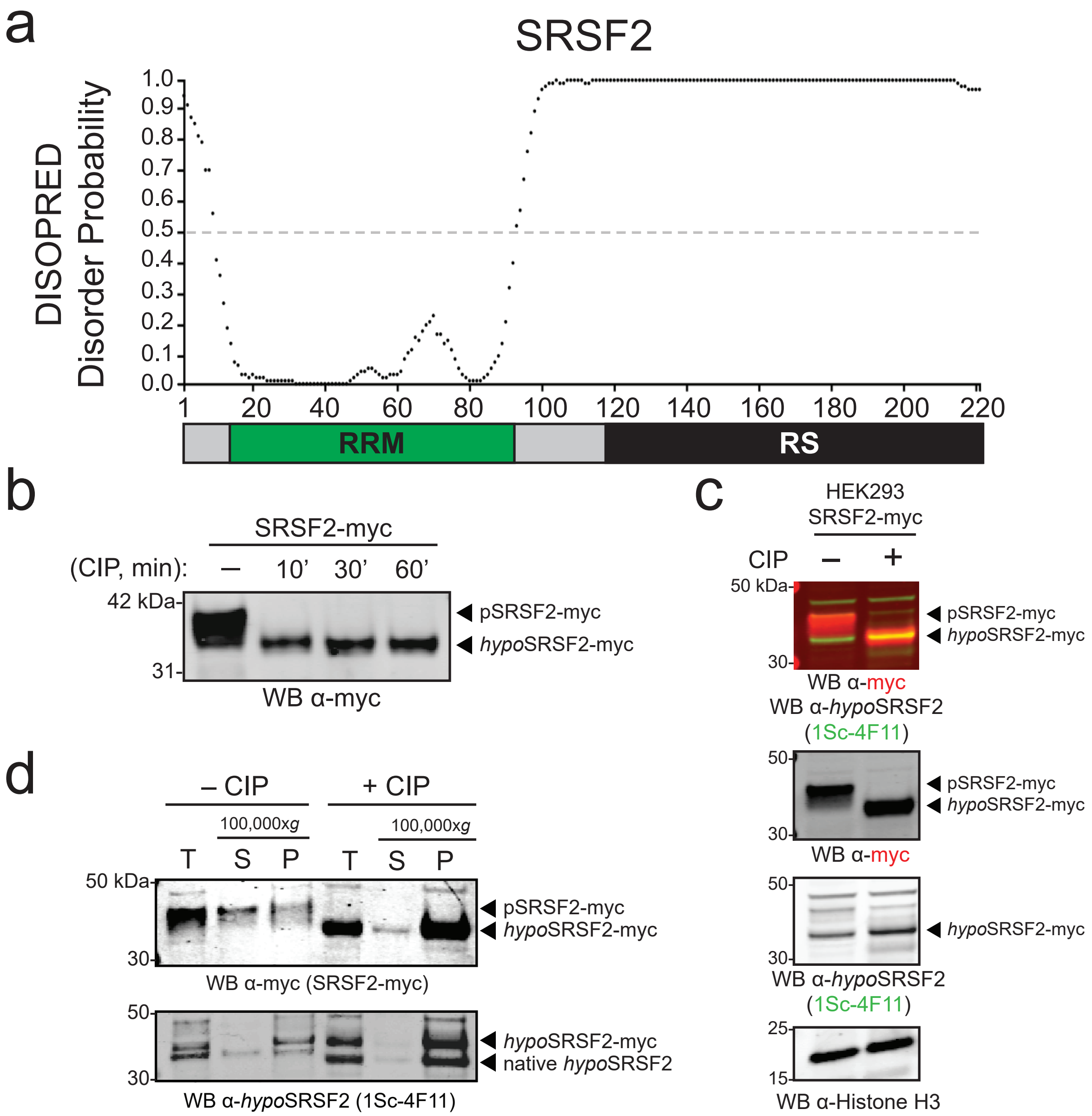

Supplemental Figure S1. The arginine-/serine-rich (RS) domain of SRSF2 is predicted to be highly disordered. The DISOPRED 3.0 algorithm predicts intrinsically disordered regions of proteins based on primary sequence alone. The protein architecture of SRSF2 below is sized to match the x-axis of the disorder plot. SRSF2 harbors an N-terminal RNA-recognition motif (RRM) domain (*green box*) and a C-terminal arginine-/serine-rich (RS) domain (*black box*). **(b)** Nucleoplasm extracts of HEK293 cells expressing recombinant SRSF2-myc protein were incubated with either distilled calf intestinal alkaline phosphatase (+CIP) for increasing time lengths (10 min, 30 min, 1 hour) or water (-CIP) or at 37°C and separated by denaturing SDS-PAGE and immunoblotted for the myc tag. **(c)** Nucleoplasm fractions of HEK293 cells transiently over-expressing SRSF2-myc were treated with either dH_2O (-) or CIP (+), separated by SDS-PAGE and immunoblotted for myc tag (*red*) and hypophosphorylated SRSF2 (*green*, hypoSRSF2) as well as Histone H3 for loading control. Increased electrophoretic mobility of SRSF2-myc was observed in +CIP treatment, along with increased labeling by hypoSRSF2-specific antibody (1Sc-4F11). **(d)** Pre-spin input (total, *T*), supernatant (soluble, *S*) and insoluble pellets (*P*) from HEK293 cells expressing recombinant SRSF2-myc were run by SDS-PAGE and western blotted for myc and hypoSRSF2. Both recombinant SRSF2 (hypoSRSF2-myc) and endogenous SRSF2 (native hypoSRSF2) labeling was observed using the hypoSRSF2 antibody. Labeling of the hypo forms of both the recombinant and endogenous sources of SRSF2 were only identified in the pellet subfraction and were increased under the +CIP condition.

Supplemental Figure S2

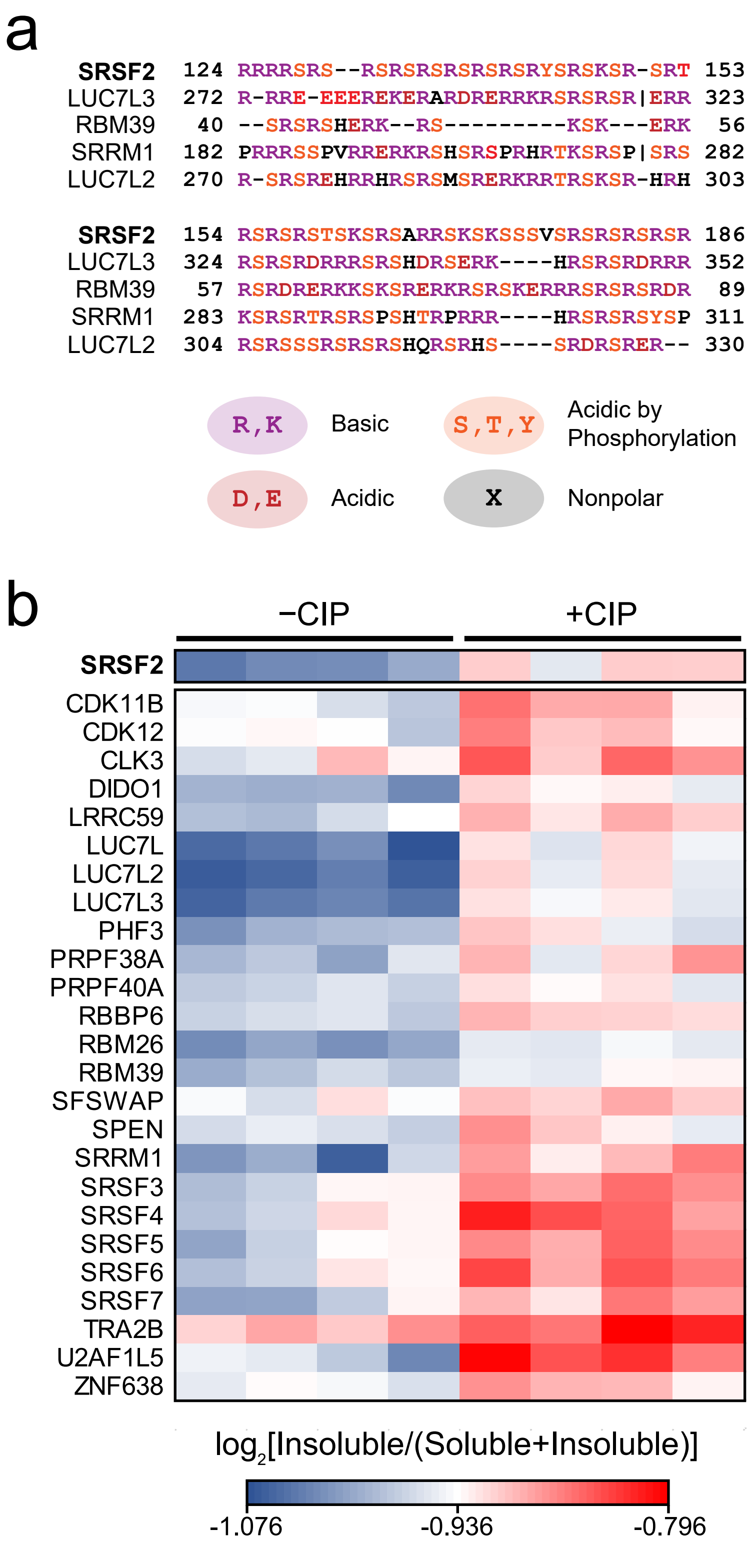

Supplemental Figure S2. SR and SR-like proteins are enriched in detergent insoluble pellet when dephosphorylated. (a) Protein BLAST of the RS domain of SRSF2 identified 193 SR and SR-like proteins. Protein sequence alignment with residues colored according to charge. Sequences were aligned and compared according to the residue charge status (basic=*purple*, acid=*red*, acidic when phosphorylated=*orange*, nonpolar=*black*). **(b)** The group of SR and SR-like proteins with significantly altered fraction insoluble values following dephosphorylation.

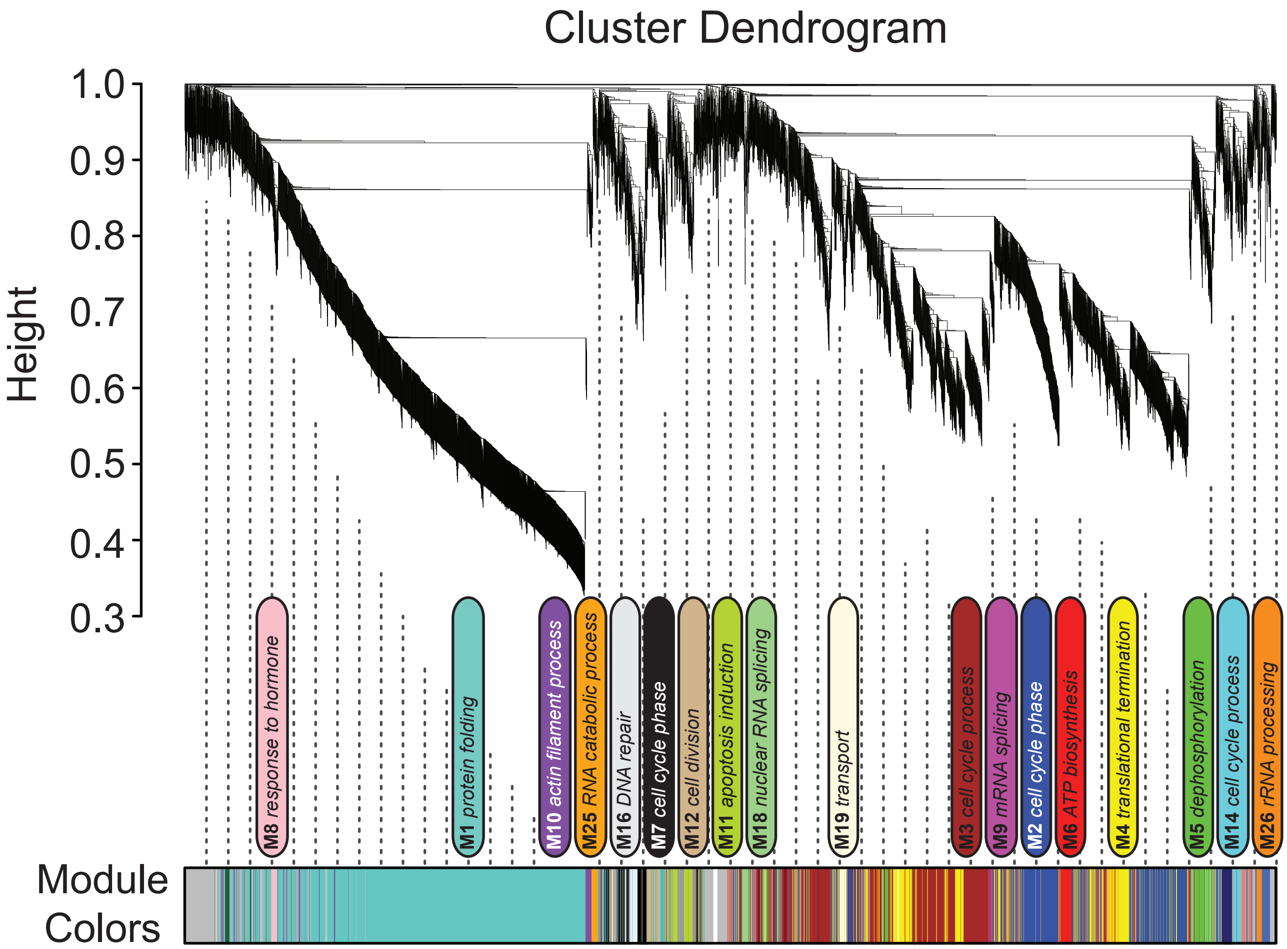

Supplemental Figure S3. The nuclear proteome was separated into discrete groups by weighted gene correlation network analyses. Weighted Gene Correlation Network Analysis (WGCNA) cluster dendrogram groups all proteins (*n*=4,120) measured by hierarchical clustering into 27 different protein modules (M1-M27). The top generalizable biological process gene ontology term was given as a title to each module.

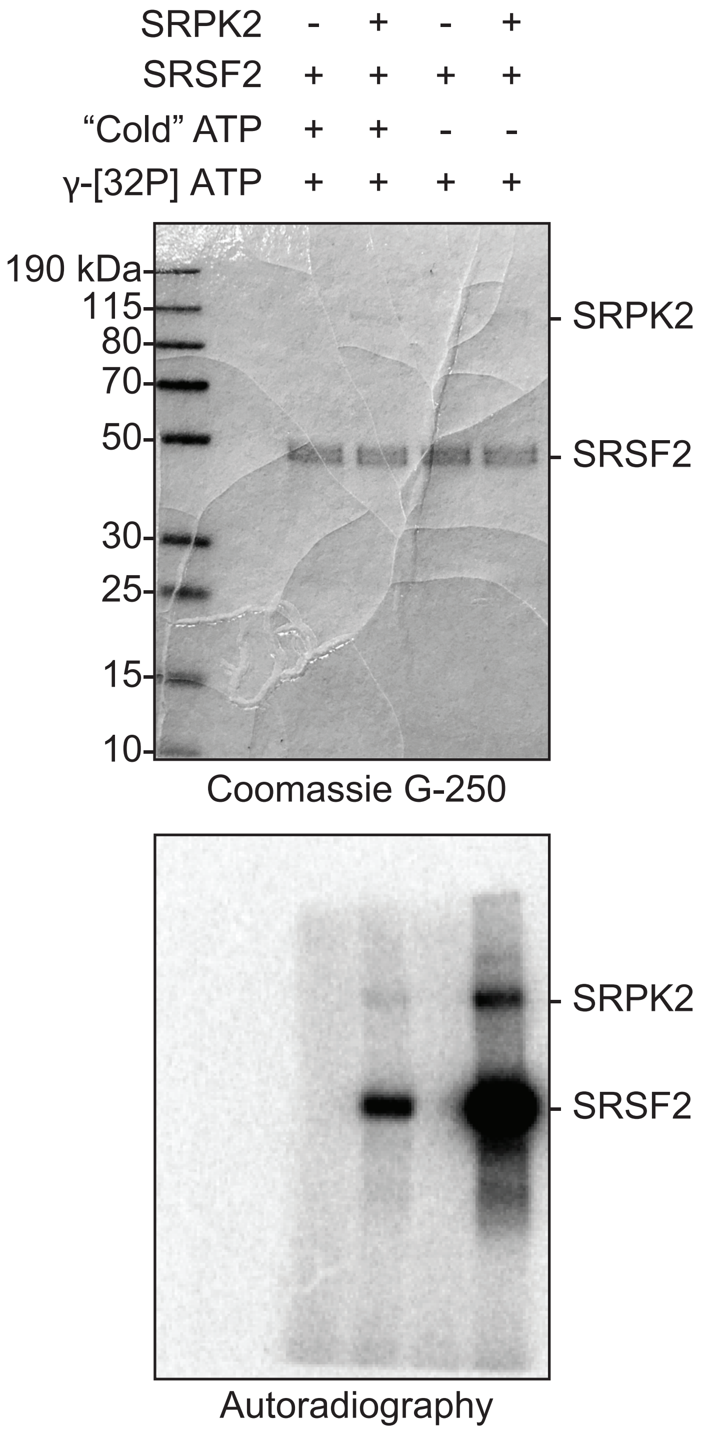

Supplemental Figure S4. Validation of *in vitro* SRPK2-SRSF2 kinase reaction. (**a-b**) Serine-/arginine protein kinase 2 (SRPK2) and SRSF2, expressed and purified from *E. coli* were mixed in the presence or lack thereof (-/+, respectively) γ–[^{32}P]ATP and incubated at 30°C for 10 min and run by denaturing SDS-PAGE or autoradiography.

Supplemental Figure S5

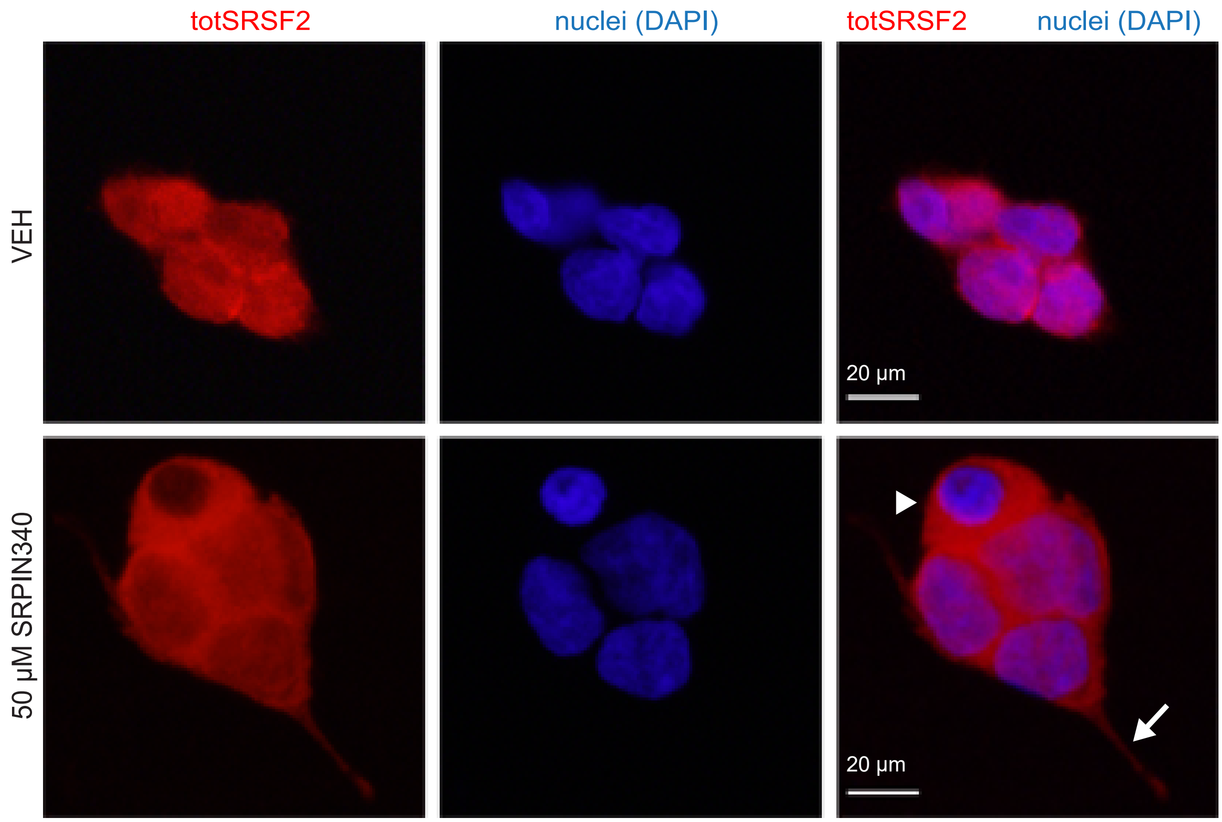

Supplemental Figure S5. Hypophosphorylated SRSF2 exhibits nuclear mislocalization phenotypes. ICC staining of vehicle (DMSO) and 50 µM SRPIN340-treated HEK293 cells for total SRSF2 (totSRSF2, *red*) and DAPI (nuclei; *blue*). We observed cells with SRSF2 cytoplasmic mislocalization (*arrowhead*) and tubule formation (*arrow*).
